# A Prototype Rapid Tool to Enhance Detection of Dementia for Aboriginal and Torres Strait Islander Peoples in Primary Care

**DOI:** 10.1002/gps.70126

**Published:** 2025-07-14

**Authors:** Huong X. T. Nguyen, Zoë Hyde, Kate Smith, Roslyn Malay, Leon Flicker, Rosie Watson, Kylie Radford, Sarah Russell, Rachel Quigley, Betty Sagigi, Edward Strivens, Adrienne Withall, Alison Timbery, Terrence Donovan, Brian Draper, Kim Delbaere, Louise Lavrencic, Robert Cumming, Jo‐anne Hughson, Bridgette J. McNamara, Dina LoGiudice

**Affiliations:** ^1^ Department of Medicine ‐ Royal Melbourne Hospital The University of Melbourne Melbourne Australia; ^2^ Western Australian Centre for Health and Ageing Medical School University of Western Australia Perth Australia; ^3^ Centre for Aboriginal Medical and Dental Health Medical School University of Western Australia Perth Australia; ^4^ Neuroscience Research Australia Sydney Australia; ^5^ School of Psychology University of New South Wales Sydney Australia; ^6^ Ageing Futures Institute University of New South Wales Sydney Australia; ^7^ College of Medicine and Dentistry James Cook University Cairns Australia; ^8^ Cairns and Hinterland Hospital and Health Service Cairns Australia; ^9^ Torres and Cape Hospital and Health Service Thursday Island Australia; ^10^ Discipline of Psychiatry and Mental Health University of New South Wales Sydney Australia; ^11^ Falls, Balance and Injury Research Centre Neuroscience Research Australia Sydney Australia; ^12^ School of Health Sciences University of New South Wales Sydney Australia; ^13^ School of Public Health University of Sydney Sydney Australia; ^14^ Centre for Epidemiology and Biostatistics University of Melbourne Melbourne Australia; ^15^ Barwon South‐West Public Health Unit Barwon Health Geelong Australia

**Keywords:** aboriginal and torres strait islander peoples, case detection, cognitive assessment, dementia, screening

## Abstract

**Introduction:**

Dementia is prevalent within Aboriginal and Torres Strait Islander communities but clients attending primary care often remain undiagnosed. This project aimed to develop a rapid dementia screen for primary care.

**Methods:**

Logistic regression was used to identify candidate items from the Kimberley Indigenous Cognitive Assessment (KICA‐Cog). The psychometric properties of different scales were assessed using receiver operating characteristic curve analysis and validated in a separate cohort.

**Results:**

Four items in the KICA‐Cog demonstrated high sensitivity (82.6%), specificity (83.2%) and area under the curve (AUC = 0.90; 95% CI: 0.87–0.94) for dementia at a cut‐off point of 7/8 out of 10. This scale has favourable psychometrics (sensitivity 87.5%, specificity 80.9%, AUC = 0.92; 95% CI: 0.85–0.98) when validated in separate cohort.

**Discussion:**

The proposed prototype tool, ready for community piloting and validation, may be useful in primary care to enable rapid cognitive screening as part of routine health care.

## Summary

- A four‐item subset of the KICA‐Cog demonstrated high sensitivity, specificity, and discriminatory accuracy for dementia identification.
- The proposed four‐item prototype scale may be useful in primary care to enable rapid cognitive screening of Aboriginal and Torres Strait Islander clients as part of routine health care.

## 1 Introduction

Dementia poses an increasing global public health challenge, driven by population ageing [1]. In Australia, dementia is three to five times more common in Aboriginal and Torres Strait Islander peoples compared to non‐Indigenous Australians [2, 3, 4]. This disparity will likely become more pronounced in the coming decades with the number of Aboriginal and Torres Strait Islander peoples aged ≥ 50 years with dementia projected to increase between 4.5 and 5.5 times from 2016 to 2051 [5]. Despite the high prevalence of dementia in Aboriginal and Torres Strait Islander communities, audits of Aboriginal primary health care services as part of the Let's CHAT (Community Health Approaches To) Dementia study revealed that only 6.3% of 763 participants aged ≥ 60 years had a diagnosis of cognitive impairment and 3.4% had a documented diagnosis of dementia [6]. These observations fall short of the dementia prevalence reported in cohort studies (> 13% for those aged ≥ 60 years) and highlight that Aboriginal and Torres Strait Islander clients attending primary care often remain undiagnosed [7].

Numerous factors contribute to the underdiagnosis of cognitive issues in primary care encompassing client, disease, clinician and systemic factors [8]. For Aboriginal and Torres Strait Islander peoples, possible barriers include stigma and cultural perceptions that may minimise memory‐related concerns, a perceived lack of culturally appropriate services, financial constraints, apprehension to engage in discussions about residential care and leaving Country, and managing competing priorities within family and communities [8, 9]. Diagnosing dementia in this population is further complicated by diverse clinical presentations, high rates of multimorbidity, and uncertainties regarding aetiology and pathophysiology, particularly in the early stages of the disease. These challenges, compounded by lack of familiarity with culturally validated cognitive assessment tools, may undermine clinicians' confidence in making a diagnosis of dementia within busy primary care settings [9]. Detection of cognitive concerns in primary care, including Aboriginal Community Controlled Health Organisations (ACCHOs), is often the first but crucial step towards further assessment. Early and accurate diagnosis of dementia provides opportunities for the proactive management of risk factors, with the potential to slow disease progression. It also facilitates access to therapeutics and interventions that may alleviate behavioural and psychological symptoms and support the maintenance of functional independence. Furthermore, timely diagnosis allows clients and family carers to access appropriate supports and plan for the future in culturally meaningful ways.

Screening tools have been demonstrated to improve detection of dementia, beyond clinician impressions [10, 11, 12]. For Aboriginal and Torres Strait Islander peoples, the 16‐item Kimberley Indigenous Cognitive Assessment (KICA‐Cog) is a culturally appropriate and validated cognitive assessment tool. Originally developed in the remote Kimberley region of Western Australia [13, 14] and validated with communities in the Northern Territory [14], the KICA‐Cog has since been adapted and validated for use in diverse contexts, including urban and regional Aboriginal communities [15], the Torres Strait [16] and First Nations peoples internationally [17, 18, 19, 20]. A 10‐item KICA‐Screen has been validated for face‐to‐face [21] and telehealth administration [22] and a carer cognitive report, the KICA‐Carer [23] has been validated for use with family members. Although these validated tools are freely available online at https://www.iawr.com.au/, and widely implemented in health and aged care policy and practice, their uptake may be hindered by limited clinician familiarity—particularly in urban areas and by administration times. Even the KICA‐Screen, which takes significantly less time to administer (5–10 min) than the comprehensive KICA‐Cog (20–25 min) and is comparable to the widely used Mini‐Mental State Examination (MMSE) [24], may not meet the rapid assessment needs of primary care clinicians. While the KICA‐Carer requires < 5 min to complete, it depends on the availability of a family or carer. This project aims to develop and validate a rapid cognitive screening tool through psychometric analysis of the KICA‐Cog ready for community‐based piloting and validation, prior to integration into routine health assessments in primary care.

## 2 Materials and Methods

### 2.1 Harmonised Dataset

The Kimberley Healthy Adults Project (KHAP) 2004–2013 [2], the Koori Growing Old Well Study (KGOWS) 2010–2018 [3], and the Dementia Prevalence Study in the Torres Strait (TSDPS) 2015–2018 [4] are Australian population ageing studies with Aboriginal and Torres Strait Islander communities. These studies collaborated closely with local community organisations and Indigenous stakeholders to ensure culturally appropriate engagement with participants. A structured interview protocol comprehensively assessed demographic, medical, and psychosocial factors, supplemented by information from family carers where available. Diagnoses were conducted by trained specialists (geriatricians, old‐age psychiatrists, general physicians) with subsequent review by a clinical panel to achieve consensus diagnoses based on the Diagnostic and Statistical Manual of Mental Disorders, Fourth Edition, Text Revision (DSM‐IV‐TR) criteria, categorising participants into normal cognition, dementia, mild cognitive impairment (MCI), or cognitive impairment not dementia (CIND) groups [25]. The CIND and MCI categories reflected cognitive decline meeting DSM‐IV‐TR criteria for cognitive or amnestic disorders, excluding psychiatric illness or intellectual disability, with minimal impact on activities of daily living (ADL). Further details on the harmonisation of these three studies are available in a separate publication [26].

### 2.2 Ethics and Governance

The KHAP, KGOWS, and TSDPS studies all adhered to the Australian National Health and Medical Research Council (NHMRC) guidelines for ethical conduct in research involving Aboriginal and Torres Strait Islander peoples and communities. Each study held ethics approval from both local Human Research Ethics Committees (HRECs) and the relevant Aboriginal health and medical research committees. The harmonisation of these studies received ethics approval from the Aboriginal Health and Medical Research Council (AH&MRC; 1362/18), the Western Australian Aboriginal Health Ethics Committee (WAAHEC; 858), the University of Melbourne HREC (12140), the Far North Queensland HREC (HREC/2020/QCH/63123–1432), and James Cook University (H9079). Additional ethics approval to validate a KICA‐based rapid cognitive screen in the Let's CHAT Dementia study cohort was granted by the AH&MRC and the University of Melbourne. This project is guided by the stewardship of the Indigenous Reference Group of the Let's CHAT Dementia study.

### 2.3 The KICA‐Cog

All three cohort studies used the KICA‐Cog, the Torres Strait adaptation of the KICA‐Cog, or the modified form (mKICA) adapted for regional and urban communities. The three iterations of the screening tool both contain 16 items that assess different domains of cognition and are scored out of a possible 39 points. The Torres Strait adaptation comprised minor modifications to wording and pictures appropriate for the Torres Strait. The mKICA contains modifications to items 1,2,4–6,8 and 9 with updated wording that is more suitable for urban‐dwelling Aboriginal and Torres Strait Islander peoples. Importantly, participants are instructed to name *any* animals (not just those you can hunt) and prompts for the verbal fluency task in item 9 are omitted in the mKICA [15]. The KICA‐Cog items are provided in Table A1.

### 2.4 Development of Short Scales

Data were managed and analysed with the statistical package Stata release 17.0 (StataCorp, College Station, TX). Descriptive statistics (mean and standard deviation [SD] for normally distributed continuous variables; median and interquartile range [IQR] for non‐normally distributed continuous variables; proportions for categorical variables) were used to summarise the data. Logistic regression was undertaken to evaluate the association between the individual items of the KICA‐Cog and outcome of (a) dementia or (b) cognitive impairment (dementia or CIND). Participants with missing data for one or more KICA‐Cog items were excluded from these analyses. Results were reported as odds ratios (OR) with 95% confidence intervals (95% CI). All KICA‐Cog components that were found to be statistically significant in univariable analyses were subsequently entered into a multivariable model, after which non‐significant items were removed through a process of backward selection. The psychometric properties of various short scales based on candidate items identified in the multivariable model were examined using receiver operating characteristic (ROC) curves and area under the curve (AUC) calculations. The sensitivity and specificity of the different short scales were reported at their optimal cut‐offs, determined using Youden's method [27]. Multivariable logistic regression was used to explore the effect of potential confounding variables such as age, sex and education. *p* values < 0.05 were considered statistically significant.

### 2.5 Validation of the Short Scales

The items identified as most sensitive to dementia and/or cognitive impairment in the KICA‐Cog were psychometrically assessed in participants from the Let's CHAT Dementia study, a stepped‐wedge cluster randomised controlled trial conducted in collaboration with 12 ACCHOs across four Australian states [9]. Comprehensive geriatric assessments (CGA) were administered to 86 participants, detailing medical conditions and measures of cognitive function including the full KICA‐Cog, Clock Test, daily function, Good Spirit Good Life quality of life tool, physical activity, psychosocial wellbeing, and collateral information from carers or family [9]. To enable comparison of comorbidities between the harmonised dataset and the Let's CHAT Dementia CGA cohort, nine conditions (stroke, diabetes, cardiac disease, renal disease, epilepsy, depression, hypertension, urinary incontinence and mobility impairment) common to both groups were calculated. Ability to perform dressing, cleaning and cooking that were assigned values of 0,1,2 based on independence, some assistance required and dependence, respectively, were summed to yield an ADL assistance score out of six. A score of 2 or more denotes dependence in at least one domain or assistance required in at least two domains of dressing, cleaning or cooking. Researchers involved in administering and scoring the KICA‐Cog received training prior to the commencement of the study to optimise inter‐rater reliability. Diagnoses for this validation cohort was assigned by two specialist geriatricians (D.L and H.N) based on clinical information, blinded to KICA‐Cog assessments and scores. Participants were independently categorised as having normal cognition, dementia or CIND in accordance with DSM‐IV‐TR criteria, consistent with the harmonised dataset. Discrepancies resolved through discussion to achieve consensus [25, 26]. The psychometric properties of the developed short scales were assessed using ROC curve analyses, as previously described.

## 3 Results

The harmonised dataset included 898 participants (KHAP wave 2 *n* = 288; KGOWS *n* = 336; TSDPS *n* = 274). There were 113 (13%) participants with a consensus diagnosis of dementia, 131 (15%) with CIND and 654 (73%) with normal cognition. The mean age of participants was 64.4 (± 9.8) years, and 60% were female. Most participants (63%) resided in remote settings and the remainder lived in urban (16%) or regional (22%) settings. The majority (71%) had some secondary schooling or higher education. After exclusion of missing data, the regression models were run on 808 participants with complete KICA‐Cog assessments with a median age of 63.0 years [IQR 59, 70] and 61% female. The mean KICA‐Cog score was 36.4 (± 3.6) in this group, including 80 participants (10%) with a diagnosis of dementia and 200 (25%) with either dementia or CIND.

Together, KICA‐Cog items 1 (orientation), 3 (orientation), 9 (verbal fluency), 10 (recall of three hidden objects), and 13 (recall of five pictures) were strongly associated with having a diagnosis of dementia, while items 1, 8 (two‐stage command), 9, 10, and 13 were more sensitive for any cognitive impairment (dementia or CIND). After excluding participants with missing data for any of the candidate KICA‐Cog items (1,3,8,9,10 or 13), ROC analyses were performed on a sample of 833 participants (10% with dementia, 15% with CIND and 75% with normal cognition) to allow for head‐to‐head comparison of the different short scales. Table 1 summarises the psychometric properties and optimal cut‐off scores of developed short scales.

**TABLE 1:** ROC analyses for short scales.

Short scales	Optimal cut‐off point of developed tools	Sensitivity (%)	Specificity (%)	AUC (95% CI)
Dementia outcome				
5‐items	10/11 out of 13	87.2	80.1	0.91 (0.88–0.94)
1/3/9/10/13				
4‐items	6/7 out of 8	62.8	91.6	0.84 (0.78–0.89)
1/3/9/10				
4‐Items	7/8 out of 10	82.6	83.1	0.90 (0.87–0.94)
1/3/9/13				
Cognitive impairment (dementia or CIND) outcome				
5‐items	10/11 out of 13	64.7	85.6	0.81 (0.78–0.85)
1/3/9/10/13				
4‐items	7/8 out of 8	69.6	76.5	0.76 (0.72–0.79)
1/3/9/10				
4‐items	8/9 out of 10	78.7	69.7	0.81 (0.77–0.85)
1/3/9/13				
5‐items	11/12 out of 14	67.6	85.8	0.82 (0.78–0.85)
1/8/9/10/13				
4‐items	8/9 out of 9	72.0	76.2	0.77 (0.73–0.81)
1/8/9/10				
4‐items	8/9 out of 11	62.3	88.5	0.81 (0.78–0.85)
1/8/9/13				

Abbreviations: AUC = area under the ROC analyses curve; CIND = cognitive impairment not dementia; ROC = receiver operating characteristic.

### 3.1 Dementia Outcome

For each one‐point increase in the total KICA‐Cog score, there was a 39% decrease in the odds of having a diagnosis of dementia (OR = 0.61, 95% CI: 0.56–0.67, *p* < 0.001). This relationship was unchanged after adjusting for age, sex, or education (see Table A2). As a five‐item scale, KICA‐Cog items 1, 3, 9, 10, and 13 had a sensitivity of 87.2% and specificity of 80.1% with cut‐point 10/11 out of 13, and AUC of 0.91 (95% CI: 0.88–0.94). In this configuration, items 10 and 13 both assess recall. To determine whether the short scales can retain discriminatory ability using only one recall item, ROC analyses were performed on two four‐item versions—one including item 10 and the other including item 13—in addition to the original five‐item scale. The first four‐item scale (1/3/9/10) had a sensitivity of 63.2% and specificity of 91.6% with cut‐point 6/7 out of 8, and an AUC of 0.84 (95% CI: 0.79–0.89). In contrast, the alternate four‐item scale (1/3/9/13) demonstrated a sensitivity of 82.6% and specificity of 83.2% with a cut‐point of 7/8 out of 10, and an AUC of 0.90 (95% CI: 0.87–0.94). The psychometric properties of the four‐item scale (1/3/9/13) were consistent when used in different age groups, males versus female participants and those with no schooling or primary schooling only versus those with secondary schooling or further education (see Table A3).

### 3.2 Cognitive Impairment Outcome

There was a 38% decreased odds of having a diagnosis of cognitive impairment (dementia or CIND) with every one‐point increase in the KICA‐Cog score (OR = 0.62, 95% CI: 0.57–0.67, *p* < 0.001). This relationship was unchanged after adjusting for age, sex, or education (see Table A2). Items 1,3,9,10 and 13 identified as sensitive for a dementia diagnosis had a sensitivity of 64.7%, specificity of 85.7% at a cut‐off of 10/11 out of 13, with an AUC of 0.81 (95% CI: 0.78–0.85) for outcome of cognitive impairment. The first four‐item scale with items 1,3,9 and 10 had a sensitivity of 69.7%, specificity of 76.7% at the cut‐point of 7/8 out of 8, with an AUC of 0.76 (95% CI: 0.72–0.79). In contrast, the alternate four‐item version, with KICA‐Cog item 13 in place of 10 (1/3/9/13), demonstrated better sensitivity of 78.7% and AUC of 0.81 (95% CI: 0.77–0.85) but slightly lower specificity of 69.7% at the cut‐off point of 8/9 out of 10.

In multivariable analyses with dementia and CIND as the dependent variable, KICA‐Cog items 1, 8, 9, 10 and 13 were retained in the model. These five items showed a sensitivity of 67.6% and specificity of 85.7% at a cut‐off of 11/12 out of 14, and an AUC of 0.82 (95% CI: 0.78–0.85). As a four‐item scale, items 1,8,9 and 10 had a sensitivity of 72.1% and specificity of 76.2% at a cut‐off of 8/9 out of 9, and an AUC of 0.77 (95% CI: 0.73–0.81) whereas items 1, 8, 9 and 13 had higher specificity (88.6%) but lower sensitivity (62.3%) at cut‐off of 8/9 out of 11, with an AUC of 0.81 (95% CI: 0.78–0.85).

The different short scales for dementia and cognitive impairment outcomes were not significantly associated with age, sex or education *p* > 0.05 (see Table A2). Univariable and multivariable logistic regression outputs for individual KICA‐Cog items for dementia and any cognitive impairment (dementia or CIND) outcomes are available in Tables A4 and A5, respectively.

Of the 86 participants with CGAs in the Let's CHAT Dementia Study, 84 had been assessed with the KICA‐Cog and were included in the analysis. The median age of participants was 74.0 years [IQR 65, 70] and 54% were female. Seventy participants (82%) had secondary schooling or further education. A consensus diagnosis of dementia was assigned to 16 (19%); CIND to 18 (21%); and normal cognition to 50 (60%) participants. The mean KICA‐Cog score for all included participants was 34.9 (± 6.0), with a median score of 37.1 (± 1.9) for those with normal cognition, 34.5 (± 3.2) for those with CIND and 28.1 (± 3.6) for those with dementia. The development (harmonised dataset) and validation (Let's CHAT Dementia cohort) were similar in terms of the proportion of male to female participants, the proportion of participants with three or more assessed medical conditions and those with a calculated ADL assistance score of two or more. The baseline characteristics of the harmonised dataset and the Let's CHAT Dementia validation cohort are presented in Table 2 for comparison.

**TABLE 2:** Baseline characteristics of development and validation cohorts.

	Strength together harmonised dataset *N* = 808	Let's CHAT dementia cohort *N* = 84	*p*‐value
Age (years) median [IQR]	63.0 [59, 70]	74.0 [65, 78]	< 0.001
Female sex	495 (61.3%)	45 (53.6%)	0.17
Secondary or further education	580 (72.5%)	69 (82.1%)	0.057
Number of medical conditions ≥ 3 (out of 9)	417 (51.6%)	47 (56.0%)	0.45
ADL assistance score ≥ 2 (out of 6)	165 (20.4%)	21 (25.0%)	0.33
Mean KICA score (SD)			
All	36.4 (3.6)	34.9 (4.3)	0.0027
Dementia	29.8 (6.6)	28.1 (3.6)	
CIND	35.4 (2.6)	34.5 (3.2)	
Normal cognition	37.4 (1.9)	37.1 (1.9)	
Cognitive status			
Dementia	80 (9.9%)	16 (19.1%)	0.01
CIND	120 (14.9%)	18 (21.4%)	0.11
Normal	608 (75.3%)	50 (59.5%)	0.0018

*Note: Comorbidity score is calculated based on the presence of nine comorbid conditions* (hearing impairment, stroke, diabetes, cardiac disease, renal disease, depression, hypertension, urinary incontinence, and mobility impairment)*; ADL assistance score (maximum total of 6 points) is based on ability to perform dressing, cooking and cleaning with each activity scored as follows: independent = 0; assistance required = 1 and dependent = 2.*

Abbreviations: CIND = cognitive impairment, not dementia; KICA‐Cog = Kimberley Indigenous Cognitive Assessment; SD = standard deviation.

### 3.3 Psychometrics of Developed Short Scales in the Validation Cohort

The psychometrics of the developed short scales are demonstrated in the validation cohort, at the previously derived optimal cut‐off point (see Table 3). The five‐item scale comprising KICA‐Cog items 1, 3, 9, 10, and 13 had a sensitivity of 93.8%, specificity of 76.5% and AUC of 0.93 (95% CI: 0.88–0.99) for a dementia diagnosis. The first four‐item scale (1/3/9/10) had a sensitivity of 81.3%, specificity of 89.7%, and an AUC of 0.94 (95% CI: 0.89–98) while the alternate four‐item scale (1/3/9/13) demonstrated a sensitivity of 87.5%, specificity of 80.9%, and an AUC of 0.92 (95% CI: 0.85–0.98).

**TABLE 3:** ROC analyses of developed short scales in the validation cohort.

Short scales	Optimal cut‐off of developed tools	Sensitivity (%)	Specificity (%)	AUC (95% CI)
Dementia outcome				
5‐items	11/12 out of 13	93.8	76.5	0.93 (0.88–0.99)
1/3/9/10/13				
4‐items	6/7 out of 8	81.3	89.7	0.94 (0.89–0.98)
1/3/9/10				
4‐items	7/8 out of 10	87.5	80.9	0.92 (0.85–0.98)
1/3/9/13				
Cognitive impairment (dementia or CIND) outcome				
5‐items	10/11 out of 13	64.7	82.0	0.85 (0.77–0.94)
1/3/9/10/13				
4‐items	6/7 out of 8	52.9	96.0	0.79 (0.69–0.88)
1/3/9/10				
4‐items	7/8 out of 10	55.9	84.0	0.84 (0.75–0.92)
1/3/9/13				
5‐items	11/12 out of 14	67.7	80.0	0.85 (0.77–0.94)
1/8/9/10/13				
4‐items	8/9 out of 9	67.7	80.0	0.77 (0.68–0.87)
1/8/9/10				
4‐items	8/9 out of 11	85.3	72.0	0.84 (0.75–0.92)
1/8/9/13				

Abbreviations: AUC = area under the ROC curve; CGA = Comprehensive Geriatric Assessment; CIND = cognitive impairment, not dementia; ROC = receiver operating characteristic.

For any cognitive impairment, the five‐item scale with items 1,3,9,10 and 13 had a sensitivity of 64.7%, specificity of 82.0% with an AUC of 0.86 (95% CI: 0.78–0.94). The first four‐item scale had a sensitivity of 52.9%, specificity of 96.0% with an AUC of 0.79 (95% CI: 0.69–0.88). In contrast, the alternate four‐item scale with KICA‐Cog item 13 in place of 10 (1/3/9/13) had a sensitivity of 55.9%, specificity of 84.0% and AUC of 0.84 (95% CI: 0.75–0.92).

Using the five KICA‐Cog items 1, 8, 9, 10 and 13, the sensitivity was 67.7%, specificity was 80.0%, and AUC was 0.85 (95% CI: 0.77–0.94) for a diagnosis of dementia or CIND. As a four‐item scale, items 1,8,9 and 10 had similar psychometric properties with sensitivity of 67.7%, specificity of 80.0%, and an AUC of 0.77 (95% CI: 0.68–0.87). In contrast, items 1, 8, 9 and 13 had higher sensitivity (85.3%) but lower specificity (72.0%) and an AUC of 0.84 (95% CI: 0.75–0.92) in this validation cohort.

## 4 Discussion

This paper investigates the psychometric properties of several short scales for rapid cognitive screening based on the KICA‐Cog. Questions assessing orientation, naming, recall and verbal fluency were highly discriminatory. KICA‐Cog items 1, 3, 9, 10, and 13 were strongly associated with having a diagnosis of dementia, while items 1, 8, 9, 10, and 13 performed best at discriminating for cognitive impairment, including dementia and CIND. Overall, the sensitivity of these short scales decreased when CIND cases were combined with dementia cases. This observation aligns with findings from other cognitive screening tools and underscores the clinical ambiguity surrounding CIND [21], as well as the limitations of brief cognitive screening tools.

**FIGURE 1:**
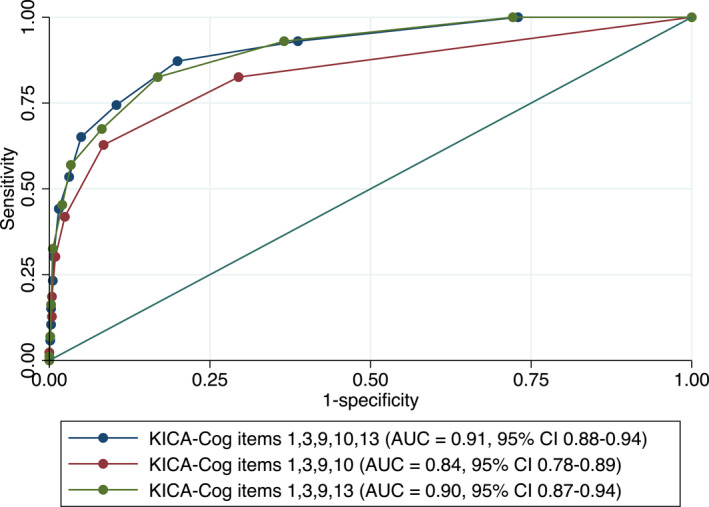
Comparison of prototype short scales. KICA items 10 (recall of three hidden objects) and 13 (free recall of five previously viewed pictures) both assess memory recall. KICA items 1,3,9,13 (green) exhibited higher AUC than the four‐item scale with item 10 (red), and comparable to the 5‐item scale (blue).

KICA‐Cog items 10 and 13 both assess memory recall, with item 10 involving recall of hidden objects and item 13 requiring free recall of previously viewed pictures. The alternate four‐item scale with item 13 exhibited higher sensitivity and discriminatory value, albeit with lower specificity, than when item 10 was used. This higher sensitivity is valuable for case identification in primary care to minimise the risk of false negatives (see Figure 1). In this way, a short tool can enhance case detection of cognitive concern that warrants further assessment.

We propose that the short scale with the best psychometrics and practical utility is the four‐item scale containing KICA‐Cog items 1,3,9 and 13 (orientation to time, orientation to place, verbal fluency and picture recall). For the detection of dementia, this prototype scale has a sensitivity of 82.6% and specificity of 83.1% at cut‐point of 7/8 out of 10 with a comparable discriminatory ability (AUC = 0.90; 95% CI: 0.87–0.94) to its five‐item counterpart. It also performed well in the validation cohort of Aboriginal and Torres Strait Islander participants from the Let's CHAT Dementia study, with a sensitivity of 87.5%, specificity of 80.9%, and AUC of 0.92 (95% CI: 0.85–0.98) for a dementia diagnosis. The proposed 10‐point scoring system for this prototype scale lends itself to rapid administration, making it particularly suitable for use in busy primary care settings. Our preliminary work suggests limited influence of age, sex and educational attainment on the proposed short scale, but further validation is required in a larger cohort (see Tables A2 and A3).

This short prototype scale did not perform as well as the original KICA‐Cog, as validated in the Kimberley (sensitivity 93.3%, specificity 94.8%, AUC = 0.98; 95% CI: 0.97–0.99), but is comparable to the adapted KICA‐Cog modified for the Torres Strait (sensitivity 81%, specificity 92%, AUC = 0.91) [16] and the KICA‐Screen when validated in Northern Queensland primary health services (sensitivity of 82.4%, specificity of 88.5% and AUC = 0.87; 95% CI: 0.77–0.97) [21]. Its psychometric properties are similar to the MMSE (sensitivity 81%, specificity 76%, AUC = 0.85; 95% CI: 0.80–0.90) and the General Practitioner Assessment of Cognition (GPCOG) when administered by general practitioners to community‐dwelling clients (sensitivity 82%, specificity 83%, AUC = 0.91; 95% CI: 0.86–0.95) [28].

Several limitations of this study should be considered. The validation cohort comprised community‐dwelling older individuals attending ACCHOs drawn from a nested cohort study. The small numbers of participants overall and those categorised as having dementia in the validation cohort limits the generalisability of the tool to the wider population. Furthermore, the consensus diagnoses assigned to participants in the validation cohort were performed by two independent specialists based on predetermined criteria, in keeping with the DSM. However, this was based on the best available information from medical records and CGA assessments and not from clinical assessment as is the gold standard. The prototype scale also demonstrated poor sensitivity (55.9%) to dementia and CIND in the validation sample and requires further evaluation to enhance its sensitivity before this tool can be implemented for relatively mild neurocognitive disorders and the early detection of cognitive decline.

The proposed four‐item prototype scale is designed for quick administration and shows promising psychometrics as a screening tool for dementia. However, further field testing and validation are required to assess its acceptability, performance and utility in primary care settings with Aboriginal and Torres Strait Islander clients. This is important in determining the optimal order and sequencing of the KICA‐Cog items, as proposed in Table 4 where item 13 is placed before item 9 in order to reduce confusion between the free recall and verbal fluency questions that both involve animals. To allow for adequate distraction between exposure to the five pictures and their recall, item 8 has been retained in the proposed prototype scale as a placeholder. However, further validation and feedback from key stakeholders including clients, clinicians in primary care and consultation with Indigenous Elders is necessary to determine how the final rapid prototype scale should be administered and scored prior to clinical implementation. Future research should also explore ways to integrate this tool into routine clinical practice with links to best‐practice guidelines to streamline processes and truncate the time lag to appropriate assessment, diagnosis, management and access to support services. One such means would be through integration with existing annual health assessments such as the Medicare Benefits Schedule (MBS) item 715 available to older Aboriginal and Torres Strait Islander people aged 55 years and over in primary care, identified as a potential enabler of high quality dementia care at the primary care interface [8].

**TABLE 4:** Proposed administration of the prototype scale.

KICA	Cognitive domain	KICA question		Score
	Picture naming	I'll show you some pictures. You tell me what they are. Remember these pictures for later on.		
		Show Boy, Dog, Billy/fire, Horse, Bicycle Point to each picture and ask, ‘What is this?’ or **Show Boy, Emu, Billy/fire, Crocodile, Bicycle. Point to each picture and ask ‘What is this?’		
		Now remember them because I'll ask you one more time.		
Item 1	Orientation week/month	Is this week pension/pay week? Or what month is it?	Not correct = 0	/1
			Correct = 1	
Item 3	Orientation place	What is the name of this community/place?	Not correct = 0	/1
			Correct = 1	
	Distraction (Item 8)	Can you point to the ceiling (or sky if outdoors) and then point to the floor?		
Item 13	Free recall ‐ 5 pictures	You remember those pictures I showed you before? What were those pictures? Tell me. Boy, emu billy/fire, crocodile, bicycle or **Boy, Dog, Billy/fire, Horse, Bicycle	0 1 2 3 4 5 0 correct = 0 1 correct = 1 2 correct = 2 3 correct = 3 4 correct = 4 5 correct = 5	/5
Item 9	Verbal fluency—naming animals (1 min)	Tell me the names of all the animals that people hunt. Time for 1 minute and can prompt e.g. ‘Keep going’, ‘Any more, e.g. in the water?’ Or Tell me the names of as many different animals as you can. We'll see how many animals you can name in 1 minute. Ready? **no prompt given and any animals allowed (not just animals you can hunt) in urban and regional settings Total No._________	0 1 2 3 0 animals = 0 1–4 animals = 1 5–8 animals = 2 9 animals or more = 3	/3
			TOTAL	/10

## 5 Conclusions

Population ageing will contribute to the rising burden of dementia experienced by Aboriginal and Torres Strait Islander communities. Timely recognition and rapid assessment in primary care is a first but crucial step towards best‐practice diagnosis and care for older Aboriginal and Torres Strait Islander peoples and their families. We propose a four‐item prototype tool based on KICA‐Cog items 1, 3, 9, and 13, intended for rapid cognitive screening in general practice as part of routine health assessments. This tool exhibits promising psychometric properties, with a sensitivity of 82.6%, specificity of 83.2%, and an AUC of 0.90 (95% CI: 0.87–0.94) for the detection of dementia but may require further development to enhance its sensitivity to CIND. Validation in an independent cohort demonstrated similar performance metrics for dementia, including a sensitivity of 87.5%, specificity of 80.9%, and an AUC of 0.92 (95% CI: 0.85–0.98). Further testing, validation, and consultation with Indigenous Elders governance groups and key stakeholders are necessary before clinical implementation.

## Conflicts of Interest

The authors declare no conflicts of interest.

## Data Availability

Data will not be made publicly available due to ethical requirements. External collaborators can apply to the KHAP, KGOWS and TSDPS steering committees for further information about individual datasets for future studies.

## Appendix

**TABLE A1:** The KICA‐Cog cognition items

Item no.	KICA‐cog parameter	Questions	Score
1.	Orientation week/month	Is this week pension/pay week? OR what month is it?	0 1
2.	Orientation season/time of year	What time of year is it now? OR what season is it now?	0 1
3.	Orientation place	What is the name of this community/place?	0 1
4.	Recognition 3 items—comb/cup/matches	*Hold up each item in turn and ask:* What do you call this? 4.1 Comb 4.2 Pannikin OR cup 4.3 Matches	0 1 0 1 0 1
5.	Use 3 items—comb/cup/matches	*Hold up each item in turn and ask:* What is this one for? a. Comb b. Pannikin OR cup c. Matches	0 1 0 1 0 1
6.	Registration—hide 3 items	Name me those things I showed you	0 1 2 3
7.	Verbal comprehension—close eyes	I'm going to ask you to do some different actions for me. Close your eyes	0 1
8.	Verbal comprehension—pointing	(Indoors) point to the ceiling and then point to the floor. OR (if outdoors) first point to the sky, and then point to the ground	0 1 2
9.	Verbal fluency—naming animals	Tell me the names of all the animals that people hunt. *Time for 1 minute, can prompt.* Total No._________ ^a^No prompt given and any animals allowed (not just animals you can hunt)	(0 animals) 0 (1–4 animals) 1 (5–8 animals) 2 (9 animals or more) 3
10.	Recall—locate 3 items	Where did I put the comb? Where did I put the matches? Where did I put the cup?	0 1 2 3
11.	Visual naming—5 pictures	I'll show you some pictures and you tell me what they are. Let's practice. Show boomerang or guitar. Point to picture & ask ‘what is this?’ Don't include in score I'll show you some more pictures and you tell me what they are. Remember these pictures for later on. Show boy, emu, billy/fire, crocodile, bicycle. Point to each picture and ask, ‘what is this?’ OR show boy, dog, billy/fire, horse, bicycle. Point to each picture and ask, ‘what is this?’	*boy, emu (or, billy/fire, crocodile, bicycle)* 0 1 2 3 4 5
12.	Executive—drawing	Look at this. Now you copy it. XOXOXO	0 1
13.	Free recall—5 pictures	You remember those pictures I showed you before? What were those pictures? Tell me.	0 1 2 3 4 5
14.	Cued recall—5 pictures	Which one did I show you before? (*One of three pictures; use boomerang page as example*)	0 1 2 3 4 5
15.	Praxis—bottle	Open this bottle and pour water into this cup	0 1
16.	Praxis ‐ comb	Show me how to use this comb.	0 1
		TOTAL	/39

*Note:* This table summarises the KICA‐Cog items, adapted from [13].

Abbreviation: mKICA = modified KICA.

^a^ Adaptations in the modified KICA for urban and regional clients.

**TABLE A2:** Short scales, adjusted for age, sex, education

KICA‐Cog items	OR (95% CI)	*p* value
Dementia outcome		
KICA‐Cog score	0.7 (0.6–0.7)	< 0.001
Adjusted for age	0.7 (0.6–0.8)	< 0.001
Adjusted for sex	0.7 (0.6–0.7)	< 0.001
Adjusted for education	0.62 (0.6–0.7)	< 0.001
5‐items	0.5 (0.4–0.6)	< 0.001
1/3/9/10/13		
Adjusted for age	0.5 (0.5–0.6)	< 0.001
Adjusted for sex	0.5 (0.4–0.6)	< 0.001
Adjusted for education	0.5 (0.4–0.6)	< 0.001
4‐items	0.4 (0.3–0.4)	< 0.001
1/3/9/10		
Adjusted for age	0.4 (0.3–0.5)	< 0.001
Adjusted for sex	0.4 (0.3–0.4)	< 0.001
Adjusted for education	0.4 (0.3–0.5)	< 0.001
4‐items	0.4 (0.4–0.5)	< 0.001
1/3/9/13		
Adjusted for age	0.5 (0.4–0.5)	< 0.001
Adjusted for sex	0.4 (0.4–0.5)	< 0.001
Adjusted for education	0.4 (0.4–0.5)	< 0.001
Cognitive impairment outcome		
KICA‐Cog score	0.61 (0.57–0.67)	< 0.001
Adjusted for age	0.65 (0.60–0.71)	< 0.001
Adjusted for sex	0.62 (0.57–0.67)	< 0.001
Adjusted for education	0.63 (0.58–0.69)	< 0.001
5‐items	0.5 (0.5–0.6)	< 0.001
1/3/9/10/13		
Adjusted for age	0.6 (0.5–0.6)	< 0.001
Adjusted for sex	0.5 (0.5–0.6)	< 0.001
Adjusted for education	0.5 (0.5–0.6)	0.003
4‐items	0.4 (0.3–0.5)	< 0.001
1/3/9/10		
Adjusted for age	0.4 (0.3–0.5)	< 0.001
Adjusted for sex	0.4 (0.3–0.5)	< 0.001
Adjusted for education	0.4 (0.3–0.5)	0.001
4‐items	0.5 (0.4–0.5)	< 0.001
1/3/9/13		
Adjusted for age	0.5 (0.5–0.6)	< 0.001
Adjusted for sex	0.5 (0.4–0.5)	< 0.001
Adjusted for education	0.5 (0.4–0.6)	0.007
5‐items	0.5 (0.5–0.6)	< 0.001
1/8/9/10/13		
Adjusted for age	0.6 (0.5–0.6)	< 0.001
Adjusted for sex	0.5 (0.5–0.6)	< 0.001
Adjusted for education	0.5 (0.5–0.6)	0.005
4‐items	0.4 (0.3–0.5)	< 0.001
1/8/9/10		
Adjusted for age	0.4 (0.3–0.5)	< 0.001
Adjusted for sex	0.4 (0.3–0.5)	< 0.001
Adjusted for education	0.4 (0.3–0.5)	0.001
4‐items	0.5 (0.4–0.5)	< 0.001
1/8/9/13		
Adjusted for age	0.5 (0.5–0.6)	< 0.001
Adjusted for sex	0.5 (0.4–0.5)	< 0.001
Adjusted for education	0.5 (0.4–0.6)	0.01

Abbreviations: CI = confidence interval; OR = odds ratio.

**TABLE A3:** Performance of short scales in different age, sex and education groups

Age	< 60 years	60–74 years	≥ 75 years
AUC (95% CI)			
5‐items	0.99 (0.98–1.00)	0.88 (0.83–0.94)	0.89 (0.84–0.95)
1/3/9/10/13			
4‐Items	0.96 (0.90–1.00)	0.83 (0.77–0.90)	0.81 (0.73–0.91)
1/3/9/13			
4‐items	1.00 (0.99–1.00)	0.87 (0.82–0.93)	0.89 (0.83–0.94)
1/3/9/13			

Sex	Male	Female
AUC (95% CI)		
5‐items	0.89 (0.83–0.94)	0.92 (0.88–0.96)
1/3/9/10/13		
4‐items	0.81 (0.73–0.89)	0.86 (0.80–0.92)
1/3/9/13		
4‐items	0.89 (0.84–0.95)	0.91 (0.87–0.95)
1/3/9/13		

Education	No schooling or primary school only	Secondary schooling or further education
AUC (95% CI)		
5‐items	0.89 (0.83–0.95)	0.90 (0.85–0.95)
1/3/9/10/13		
4‐items	0.85 (0.78–0.93)	0.78 (0.70–0.86)
1/3/9/13		
4‐items	0.88 (0.82–0.94)	0.90 (0.85–0.95)
1/3/9/13		

*Note:* AUC = area under the curve.

**TABLE A4:** Logistic regression outputs for dementia outcome

Item no.	Scoring	KICA‐Cog parameter	Univariable regression		Multivariable regression	
			OR (95% CI) *N* = 808	*p*‐value	OR (95% CI)	*p*‐value
1	incorrect	Orientation week/month	15.2 (8.5–27.3)	< 0.001	6.2 (2.7–14.4)	< 0.001
2	incorrect	Orientation season/time of year	6.7 (3.9–11.4)	< 0.001		
3	incorrect	Orientation place	38.4 (12.3–120.0)	< 0.001	15.4 (2.3–104.3)	0.005
4	incorrect	Recognition 3 items: comb	28.3 (2.9–275.6)	0.004		
	incorrect	Cup	—			
	incorrect	Matches	—			
5	incorrect	Use 3 items: comb	—			
	incorrect	Cup	—			
	incorrect	Matches	18.6 (1.7–207.9)	0.02		
6	incorrect	Registration: Hide 3 items	1	< 0.001		
	incorrect		11.1 (5.9–21.0)			
	incorrect		54.8 (14.5–206.8)			
	incorrect		38.4 (9.6–153.8)			
7	incorrect	Verbal comprehension: Close eyes	18.6 (1.7–207.9)	0.02		
8	incorrect	Verbal comprehension: Pointing	1	0.001		
	1 incorrect		4.5 (1.4–15.1)			
	2 incorrect		8.7 (2.9–26.7)			
9	> 9 animals	Verbal fluency: Animals	1	< 0.001		0.002
	5‐8 animals		4.1 (2.4–7.1)		1.6 (0.8–3.2)	
	1‐4 animals		38.7 (17.0–88.1)		8.3 (2.4–28.4)	
	0 animals		10.1 (0.9–115.2)		0.06 (0.001–3.1)	
10	0 incorrect	Recall: Locate 3 items	1	< 0.001		0.001
	1 incorrect		12.4 (6.1–24.9)		5.0 (2.0–12.4)	
	2 incorrect		9.5 (4.4–20.5)		3.6 (1.3–9.7)	
	3 incorrect		31.8 (12.5–80.5)		2.6 (0.5–14.1)	
11	0 incorrect	Visual naming: 5 pictures	1	0.006		
	1 incorrect		3.2 (1.4–7.5)			
	2 incorrect		4.2 (0.8–22.2)			
	3 incorrect		—			
	4 incorrect		—			
	5 incorrect		—			
12	incorrect	Executive: Drawing	5.3 (2.7–10.6)	< 0.001		
13	0 incorrect	Free recall: 5 picture names	0.0080 (0.0020–0.032)	< 0.001		< 0.001
	1 incorrect		3.2 (0.68–15.3)		2.2 (0.5–11.0)	
	2 incorrect		15.3 (3.5–67.6)		9.4 (2.1–43.0)	
	3 incorrect		25.8 (5.6–120.2)		7.2 (1.4–37.7)	
	4 incorrect		126.5 (26.0–616.4)		59.9 (11.2–321.2)	
	5 incorrect		227.2 (49.4–1043.9)		27.1 (4.8–152.1)	
14	0 incorrect	Cued recall: 5 pictures	1	< 0.001		
	1 incorrect		5.8 (2.9–11.4)			
	2 incorrect		16.0 (5.7–44.8)			
	3 incorrect		32.0 (7.7–132.7)			
	4 incorrect		24.0 (6.5–88.5)			
	5 incorrect		64.1 (7.0–586.2)			
15	incorrect	Praxis: Bottle	38.3 (4.22–346.7)	0.001		
16	incorrect	Praxis: Comb	9.3 (1.3–67.0)	0.03		

**TABLE A5:** Logistic regression outputs for dementia and CIND outcome

	Scoring	KICA‐Cog parameter	Univariable regression *N* = 808		Multivariable regression	
Item no.			OR (95% CI)	*p*	OR (95% CI)	*p*
1	incorrect	Orientation week/month	5.8 (3.3–10.0)	< 0.001	2.1 (1.0–4.3)	0.04
2	incorrect	Orientation season/time of year	4.0 (2.5–6.4)	< 0.001		
3	incorrect	Orientation place	11.4 (3.7–34.9)	< 0.001		
4	incorrect	Recognition 3 items: comb	—			
	incorrect	Cup	—			
	incorrect	Matches	—			
5	incorrect	Use 3 items: comb	—			
	incorrect	Cup	—	—		
	1 incorrect	Matches	6.1 (0.6–68.0)	0.1		
6	0 incorrect	Registration: Hide 3 items	1	< 0.001		
	1 incorrect		5.7 (3.2–10.3)			
	2 incorrect		46.6 (6.0–361.5)			
	3 incorrect		9.1 (2.3–35.5)			
7	1 incorrect	Verbal comprehension: Close eyes	—			
8	0 incorrect	Verbal comprehension: Pointing	1	< 0.001	38.4	0.009
	1 incorrect		7.5 (2.3–24.7)		4.5 (1.1–18.0)	
	2 incorrect		18.4 (4.0–83.8)		7.2 (1.3–38.4)	
9	> 9 animals	Verbal fluency: Animals	1	< 0.001		< 0.001
	5‐8 animals		5.1 (3.5–7.4)		3.5 (2.3–5.3)	
	1‐4 animals		31.2 (11.7–83.1)		9.4 (2.9–30.2)	
	0 animals		11.5 (1.0–128.7)		3.6 (0.1–120.9)	
10	0 incorrect	Recall: Locate 3 items	1	< 0.001		< 0.001
	1 incorrect		9.4 (4.8–18.6)		4.7 (2.1–10.4)	
	2 incorrect		5.0 (2.5–10.0)		2.7 (1.1–6.3)	
	3 incorrect		11.2 (4.3–29.2)		1.3 (0.3–6.0)	
11	0 incorrect	Visual naming: 5 pictures	1	0.006		
	1 incorrect		3.0 (1.5–5.9)			
	2 incorrect		4.4 (1.0–5.9)			
	3 incorrect		—	—		
	4 incorrect		—	—		
	5 incorrect		—	—		
12	incorrect	Executive: Drawing	4.5 (2.4–8.4)	< 0.001		
13	0 incorrect	Free recall: 5 picture names	1	< 0.001		< 0.001
	1 incorrect		1.9 (1.1–3.3)		1.5 (0.8–2.6)	
	2 incorrect		4.5 (2.5–7.9)		2.8 (1.5–5.3)	
	3 incorrect		10.0 (5.1–19.6)		3.7 (1.7–7.9)	
	4 incorrect		45.3 (15.6–131.9)		24.5 (7.9–76.0)	
	5 incorrect		65.1 (24.7–171.9)		10.5 (3.4–32.5)	
14	0 incorrect	Cued recall: 5 pictures	1	< 0.001		
	1 incorrect		6.8 (3.8–12.3)			
	2 incorrect		9.1 (3.1–26.7)			
	3 incorrect		14.5 (3.0–70.6)			
	4 incorrect		6.2 (1.7–22.3)			
	5 incorrect		16.6 (1.8–149.5)			
15	incorrect	Praxis: Bottle	—			
16	incorrect	Praxis: Comb	3.1 (0.4–21.9)	0.3		
